# Modifiable Temporal Unit Problem (MTUP) and Its Effect on Space-Time Cluster Detection

**DOI:** 10.1371/journal.pone.0100465

**Published:** 2014-06-27

**Authors:** Tao Cheng, Monsuru Adepeju

**Affiliations:** SpaceTimeLab, Department of Civil, Environmental and Geomatic Engineering, University College London, Gower Street, WC1E 6BT London, the United Kingdom; University of Warwick, United Kingdom

## Abstract

**Background:**

When analytical techniques are used to understand and analyse geographical events, adjustments to the datasets (e.g. aggregation, zoning, segmentation etc.) in both the spatial and temporal dimensions are often carried out for various reasons. The ‘Modifiable Areal Unit Problem’ (MAUP), which is a consequence of adjustments in the spatial dimension, has been widely researched. However, its temporal counterpart is generally ignored, especially in space-time analysis.

**Methods:**

In analogy to MAUP, the Modifiable Temporal Unit Problem (MTUP) is defined as consisting of three temporal effects (aggregation, segmentation and boundary). The effects of MTUP on the detection of space-time clusters of crime datasets of Central London are examined using Space-Time Scan Statistics (STSS).

**Results and Conclusion:**

The case study reveals that MTUP has significant effects on the space-time clusters detected. The attributes of the clusters, i.e. temporal duration, spatial extent (size) and significance value (p-value), vary as the aggregation, segmentation and boundaries of the datasets change. Aggregation could be used to find the significant clusters much more quickly than at lower scales; segmentation could be used to understand the cyclic patterns of crime types. The consistencies of the clusters appearing at different temporal scales could help in identifying strong or ‘true’ clusters.

## Introduction

In recent years, the advancement in geographical data collection techniques (e.g. Computer Aided Dispatch Systems (CAD), portable sensors etc.) has brought about exponential growth in the availability of geographic data at small space and time scales. This trend of data availability is now observed in many application domains including criminology, epidemiology, and transport, to mention but a few. The time stamp in these datasets provides opportunities to mine intrinsic properties of spatial events in relation to time. Hence, attention is shifting from purely spatial analysis to space–time analysis. Research efforts are now focussing on developing techniques to mine the space-time complexities within the datasets in order to further understand the dynamics underlying geographic events [1], [2].

Observations of discrete geographic data are usually made at point locations, but are often aggregated into areal units for various reasons, such as confidentiality of individual records, data summary or to fit into an existing zoning system (e.g. districts, service areas, police beats etc.). Spatial aggregation however, requires consideration of problems such as the Modifiable Areal Unit Problem (MAUP) and the ecological fallacy, which have been widely discussed in the literature [3]–[6]. Recently, the term MTUP (Modifiable Temporal Unit Problem) has been mentioned in a number of studies in analogy to MAUP [7], [8], with major focus on temporal aggregation (scales) and its effects on statistical inference [9]–[13]. However, other issues relating to the temporal dimension, such as the manner in which the temporal dimension is divided (segmentation) or adjustments to the temporal extent (boundary) of a time series, have received less attention.

Analogous to the zonation effect in the spatial dimension [14], temporal segmentation may be viewed as the situation whereby the analyst is open to a number of choices as to how the temporal dimension can be discretised into temporal units. Commonly used implementations of segmentation in large databases were examined in [15], and found to often produce disparate results. One important factor affecting the frequency distribution of a segmented dataset is the selection of the starting phase of temporal segmentation. It was further demonstrated that the selection of the starting phase of temporal segmentation influences the estimation of regression model parameters [8]. In discrete data segmentation, for example, mid-night or mid-day may be considered as the starting point of daily observations, while weekly aggregation may start from Sunday or Monday. In any case, the basic statistical estimates such as mean, variance and so on are bound to change [16].

The boundary problem is a concept mostly associated with the spatial dimension [17]. However, it was argued that the boundary problem occurs not only in horizontal boundaries but also in vertically drawn boundaries such as time, depth and temperature [18]. In temporal data, the boundary is the temporal frame within which observations of a process are made. Adjusting the frame is synonymous with adjusting the temporal boundary within which the events are bounded. However, as [19] argues, human activities are rarely bounded in this way, but extend in space and time. In general, estimation of certain variables in an analysis may depend directly or indirectly on the temporal length of the dataset.

Despite growing interest in the analysis of dynamics in geographical datasets in space and time simultaneously, issues relating to the spatial dimension continue to receive attention while their temporal counterparts are largely ignored. In geographical cluster analysis for example, a number of studies have investigated how the spatial aggregation (a component of MAUP) of spatiotemporal datasets affects the results of cluster detection [20]–[24]. Studies related to the MTUP have exclusively focused on the impacts of temporal aggregation on statistical inference in either purely temporal or spatiotemporal data analysis [12], [25]–[27]. The joint impacts of both spatial and temporal scales on space-time data analysis have only been examined in a few studies to date [28]–[29], but not a single study has examined the effects of temporal aggregation on cluster detection, let alone segmentation or boundary effects. Recently, Kwan argued that analytical results could be different for different delineations of contextual units even if everything else is the same [30]. This problem is referred to as the uncertain geographic context problem (UGCoP). The spatial uncertainty and dynamics of geographic context associated with the UGCoP greatly complicate any examination of the effect of contextual influences on health. We hope the work presented here will help us to understand the UGCoP better if not yet fully tackle the problem.

This paper investigates the impacts of not only temporal aggregation but also temporal segmentation and boundaries, which we will formally define as the three components of the MTUP in the next Section. These effects will be examined in the context of spatiotemporal cluster detection using space-time scan statistics (STSS). In the case study, STSS is used to detect clusters in three crime types (“*burglary-dwellings*”, “*theft-of-shoplifting*” and “*violence-against-persons*”) in the London Borough of Camden. Each of the three temporal effects (aggregation, segmentation and boundary) is examined individually by comparing the attributes of the detected clusters under each temporal effect.

## Temporal Effects - Modifiable Temporal Unit Problem (MTUP)

Here we define the temporal effects in analogy to the spatial effects of MAUP.

### 2.1 Temporal Aggregation Effect

Temporal scale corresponds to spatial scale (resolution) in the temporal dimension. It refers to the regularly spaced unit of time observation, which can be a minute, a day, or a week, etc. Temporal aggregation is a process that converts the observations from a fine interval into a coarse interval. Temporal aggregation is needed for various reasons, which include, for example, closing gaps in the data, data summary and reduction of data size for ease of processing. When data are not recorded at regular time intervals, adjustment is needed to convert the data into regular intervals for analysis. For example, a crime may occur in a geographic region at any time, usually being recorded to the nearest second. To make meaningful analysis, such irregularly recorded data will often be converted into measures at regular time intervals either hourly or daily (see more details in Section 3.2).

There are various forms of temporal aggregation [15] but the basic form involves discretisation of time frame from a detailed interval into a coarse one, where the number of events (e.g. the number of crimes) within each time interval is summed and reported as a single value. Summation could be replaced with averaging or taking the maximum of the number of events within the original intervals. By aggregating the data from a higher temporal scale to a lower one (e.g. from daily to weekly) the small cyclic temporal trends (low frequency variations) in data are automatically adjusted. The basic statistical estimates such as variance and correlation coefficients are affected due to the change in the number of resulting intervals [26].

**Figure 1:**
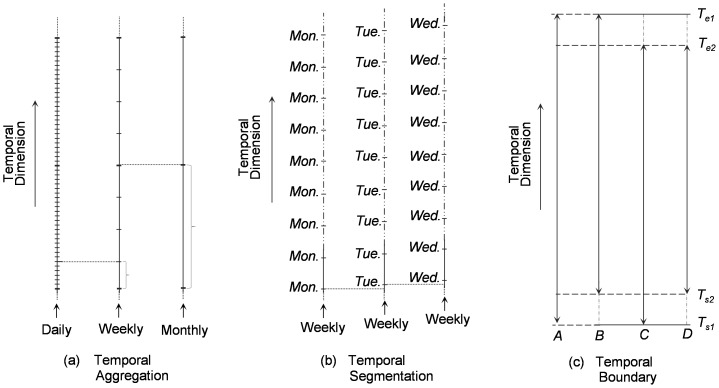
Modifiable Temporal Unit Problem (MTUP) (a) Temporal aggregation (b) Temporal Segmentation (c) Temporal boundary.

Figure 1a illustrates the aggregation of a daily scale to lower scales (weekly and monthly). The aggregation first splits the data into different intervals; the events within each interval are then summed. Thus aggregation divides the data into a coarse interval, using the data from the fine intervals. As the scale becomes smaller, the number of intervals within the temporal frame is reduced.

### 2.2 Temporal Segmentation Effect

Temporal segmentation can be likened to the zoning effect of the MAUP where the result of spatial analysis varies depending on the adoption of different zoning patterns. Similarly, in purely temporal or spatiotemporal analysis, the continuous time frame is usually divided into chunks of temporal segments, i.e. it is discretised into temporal units (partitions/portions). The temporal segmentation is usually carried out at regular intervals, with data for a day, a week, a month, or a year, etc. However, the segmentations could be different if the starting points of the intervals are different. For example, a weekly segmentation for a time frame of daily crime counts may begin on Sunday and end on the following Saturday. Equally, the segmentation could start on Monday and end on the following Sunday. A series of segmentations can be generated from a single time frame by simply varying the starting point of the temporal intervals. Figure 1b illustrates the division (partition) of a daily time frame into weekly segmentations (partitions), varying the first day of the week (Monday, Tuesday, and Wednesday). With segregation, the fine-scale (daily) data is portioned into the frame of the coarse (weekly) temporal intervals (all at same scale). The sample counts and summation of each (weekly) interval at different segmentations might change due to different starting point of the intervals. So aggregation is involved somehow in the segmentation, but aggregation emphases the scale change (from fine to coarse), while segmentation emphases the partition of the data during the process of scale change.

### 2.3 Temporal Boundary Effect

In purely spatial or spatiotemporal analysis, the term “boundary” is exclusively used to denote an arbitrary line drawn around a geographical area indicating its extent. The “boundary effect” refers to the impact that the way in which a boundary is drawn has on both the identification of the spatial distribution and the estimation of the statistical parameters of the underlying spatial process [17], [31], [32]. Here, we extend the same concept to the temporal dimension by identifying the start and end points of a time series as its temporal extent or boundary. By altering the temporal length of a space-time process, the sample counts and estimates of mean and variance would be altered. Figure 1c shows a time frame *A* with an original start (*T_s1_*) and end point (*T_e1_*). By adjusting the boundary, new temporal boundaries *B*, *C* and *D* can be obtained, with *B*, *C* and *D* bounded by [*T_s2_*, *T_e1_*], [*T_s1_*, *T_e2_*] and [*T_s2_*, *T_e2_*], respectively.

## Methods

In this study, we investigate the three MTUP effects defined in Figure 1 on space-time cluster detection by using a case study of crime pattern analysis. In the following subsections, we will first introduce the principle of space-time scan statistics (STSS), then present the case study area and the dataset, and finally describe the workflow of the experiment.

### 3.1 Space-Time Scan Statistics (STSS)

Space-Time Scan Statistics (STSS) is an extension of the popular Spatial Scan Statistics (SSS), a geographical cluster detection technique, which was originally used in the field of epidemiology for disease outbreak detection [33]. SSS was developed to overcome the limitations of the Geographical Analysis Machine (GAM) in identifying the optimal size (which is also part of the MAUP) and the significance of identified clusters [34]. STSS has so far been applied in a few domains including criminology [35], public health [36] and forestry [37]. Generally, the technique is used to investigate whether an observed cluster of events has occurred by chance, assuming that the events are distributed uniformly across the geographical region with no space-time interaction.

In operation, the technique scans through the study area using a large set of overlapping geographic windows moving across space and time [36]. Each of the scanning windows has a shape (e.g. cylinder), a base centroid (*x*, *y*), a radius *r* and time length *t*. The number of cases within the window is counted and compared with the expected count. The size of the cylinder (in space and time) is increased systematically to generate a large number of cylinders with all parameters evaluated at each instant. Considering all the cylinders together, the one with highest likelihood ratios (the ratio of observed cases with the expected value) are marked as primary candidates for true clusters. The statistical significance (p-value) of the marked cylinder is tested by random permutation (Monte Carlo replication). The p-value is calculated by dividing the count of replicas that have higher likelihood ratios than the marked cylinder with the total count of the replicas. In most cases, the p-values are compared with a threshold value α (0.05 or 0.001) in order to conclude that the space-time clusters are statistically significant (i.e. likely to be a true cluster).

A variety of scan statistical models exist, designed for application to different data types. For count data, the Poisson [33] and Space-Time Permutation scan statistics [36] are two models generally used, and are based on the null hypothesis of complete randomness in space time. For discrete data such as crime counts, Space-Time Permutation Scan Statistics (STPSS) is the most appropriate. STPSS is implemented in SaTScan software [38], and will be used for the case study described below.

### 3.2 Dataset

**Figure 2:**
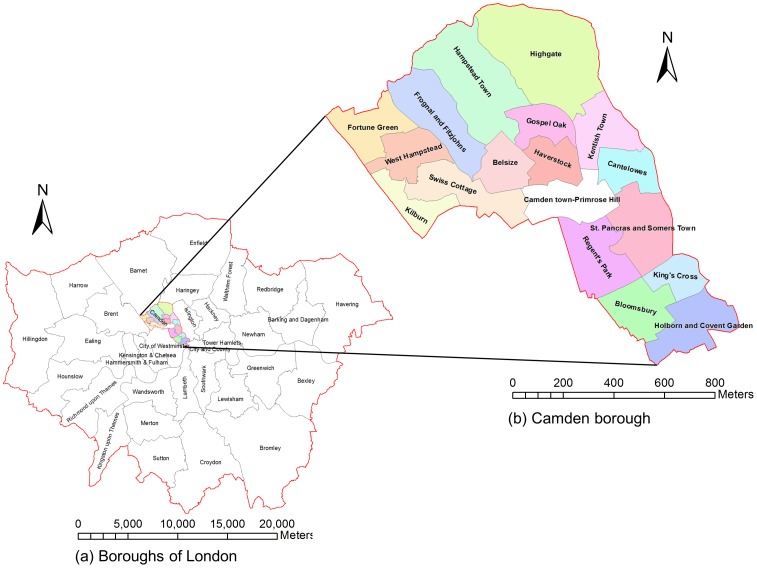
The study Area.

The data of reported crime in the London Borough of Camden is used in this study. The Borough of Camden is one of the inner boroughs of London City. It has an approximate area of 22 km^2^ with eighteen administrative wards. According to UK 2011 Census, Camden's usual resident population was 220,338 with the highest proportion of residents (27%) located in the 30–44 age band. The Borough features contrasting geo-demographic settings ranging from open space like Hampstead Heath, to very busy areas like Camden Town and Covent Garden (Fig. 2). These areas are sites of attraction to tourists and leisure seekers.

All reported crimes within London are recorded in the Metropolitan Police Service Computer Aided Dispatch (CAD) system. Between 1^st^ March 2011 and 31^st^ March 2012, a total of 28,686 records of committed crimes of different types were recorded in the database for Borough of Camden. Each data point is geocoded to the centroid point of a 250 m by 250 m grid and recorded to nearest second. Three crime types - “burglary-dwellings”, “theft-of-shoplifting” and “violence-against-persons” - were chosen in order to examine whether MTUP has different effects on different crime types. The dataset consists of 2,160, 1,072 and 3,323 records of these three crime types, respectively. As part of the data pre-processing, the temporal scale of the dataset was aggregated to a daily scale, given that the number of crimes at the original scale is too sparse for meaningful analysis.

### 3.3 The Workflow of the Experiment

**Figure 3:**
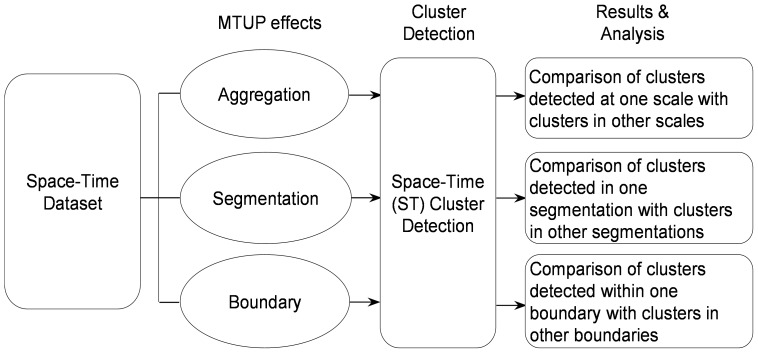
Our experimental approach to examining the MTUP effects on space-time cluster detection.

Figure 3 illustrates the workflow of the experiment. The first step involves preparing the dataset for testing each of the temporal effects, i.e. converting (temporally) irregularly recorded data (at any time of the day recorded to the nearest second) into a regular interval (daily count of observations). Intervals within which no observation was recorded are assigned the value 0.

The second step is to implement the process of adding the MTUP effects. For the temporal aggregation effect, we generated data at weekly and monthly intervals from the daily dataset generated in Step one. For the temporal segmentation, we generated seven weekly datasets by dividing the daily data in Step one into weekly units (portions) with different starting day of the week (i.e. Monday, Tuesday, Wednesday and so on); the temporal boundary effect was then examined by adjusting the length of the study period. Here, three new study periods were generated from the original study period by removing 1-month of data from the beginning, or/and the end.

The third step is to carry out the cluster detection of crime hotspots. Here the retrospective space-time permutation probability model is chosen in SaTScan (Kulldorff, 2010) with a maximum spatial cluster size equal to 50% of the at-risk population (maximum circle size of 1000 metre radius). The maximum temporal cluster size is equal to 50% of the study period, with a maximum of 999 Monte Carlo replications, and ‘No geographic overlap’ as the criteria for reporting secondary clusters and statistical significance (p-value) threshold of 0.05 (i.e. we are only interested in clusters with p-value less or equal to 0.05).

The fourth step is to compare the clustering results. To compare the results, three basic cluster attributes were used along with the count (number) of detected clusters. These attributes are; the temporal duration, spatial extent (size) and the statistical significance (p-value). The spatial locations of the cluster centroids (x, y) were used to identify the same clusters under separate analyses. Two Clusters will be considered to be the same, if the distance between their centroid points is within 250 metres.

## Results and Discussion

The textual description of clusters, as reported by SaTScan™, contains information such as the spatial location, radius, start and end dates of the cluster, number of observed and expected crime counts inside the clusters and the statistical significance value (p-value). This information is usually imported into GIS environment for proper visualisation.

### 4.1 Impacts of Temporal Aggregation

**Figure 4:**
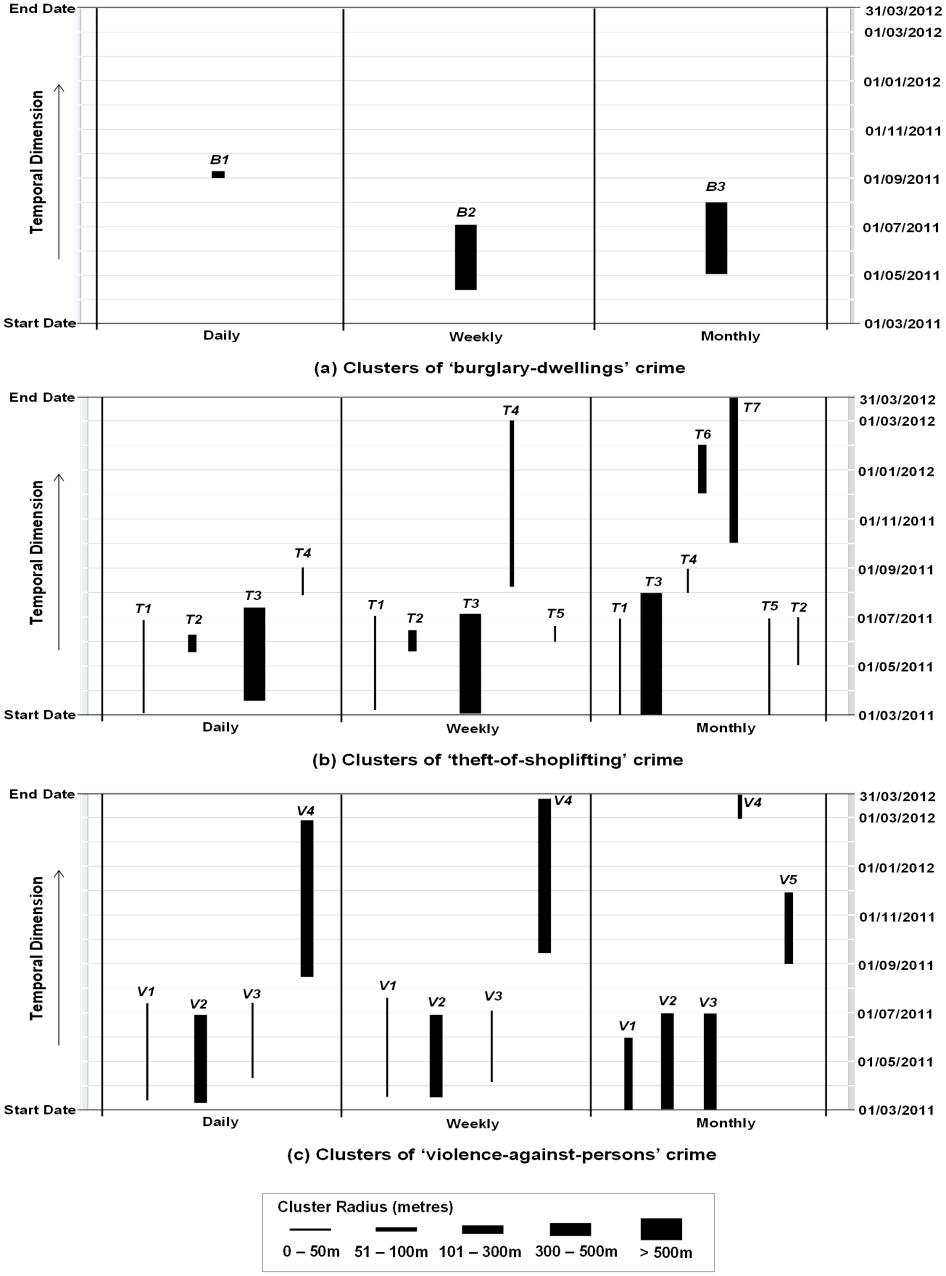
Temporal Aggregation Effects (a) “Burglary-dwellings” crime clusters (b) “Theft-of-shoplifting” crime clusters (c) “Violence-against-persons” crime clusters.

For each of the three crime types, the clusters detected at each temporal scale (i.e. daily, weekly and monthly) are placed side-by-side for comparison. The clusters are identified by their spatial locations, i.e. the centroid of the cluster (x, y). Figures 4a, 4b and 4c show the clusters detected in “burglary-dwellings”, “theft-of-shoplifting” and “violence-against-persons” datasets respectively. The spatial and temporal extents are represented by the width and height of the clusters respectively. The clusters are labelled numerically to match the same cluster detected at different scales, and the order of their appearance reflects the level of significance changing from higher to lower (all are significant at the 0.05 level).

For the “burglary-dwellings” crime (Figure 4a), only one cluster was detected at each of the three scales. The three clusters were identified as different from each other based on their spatial location and hence have different labels (i.e. *B1*, *B2*, *B3*). Moreover, their temporal duration, spatial extent (size) and statistical significance (p-values) are different (See Table S1-a in Appendix S1).

In Figure 4b, four clusters (labelled *T1*, *T2*, *T3*, and *T4*) were detected for the “theft-of-shoplifting” crime type at the daily scale, with significance decreasing from cluster *T1* to *T4*; another cluster (Cluster *T5*) was detected at the weekly scale, with a lower p-value than the other four clusters; and a further two clusters (*T6* and *T7*) were detected at the monthly scale, with p-value lower than Clusters *T1*, *T3*, *T4*, and higher than Clusters *T5* and *T2*. The four clusters (*T1–T4*) are identified at all scales with *small variation in p-values and temporal durations* but their spatial extents remain generally stable at the three scales (see Table S2-a in Appendix S1). The consistency of these 4 clusters suggests strong hotspots in these areas.

Similar patterns are shown for the “violence-against-persons” crime with 4 clusters (*V1*, *V2*, *V3* and *V4*) detected at all three scales (Figure 4c), and another cluster *V5* detected at the monthly scale. These 4 clusters display *small variations in p-values and temporal durations* at each scale, with the exception of *V4*, which has a much shorter duration at the monthly scale. Again, we can say that these 4 clusters (*V1–V4*) are strong hotspots in these areas. Further details of these clusters can be found at Table S3-a in Appendix S1.

The result demonstrates that a change in the temporal aggregation scale of the dataset affects the temporal duration, size and significance of the clusters. The effect on the “burglary-dwellings” crime type results in three different clusters being detected at different scales, but the effect on both the “theft-of-shoplifting” and “violence-against-persons” crimes types is less strong. Clusters at fine scales can equally be detected at a coarse scale, and a coarse scale tends to detect more clusters. This suggests that temporal aggregation does not reduce the power of STSS to detect clusters, though not all the clusters are significant at fine scales. The clusters appearing at all scales should be considered “true” clusters that warrant attention.

### 4.2 Impacts of Temporal Segmentation

**Figure 5:**
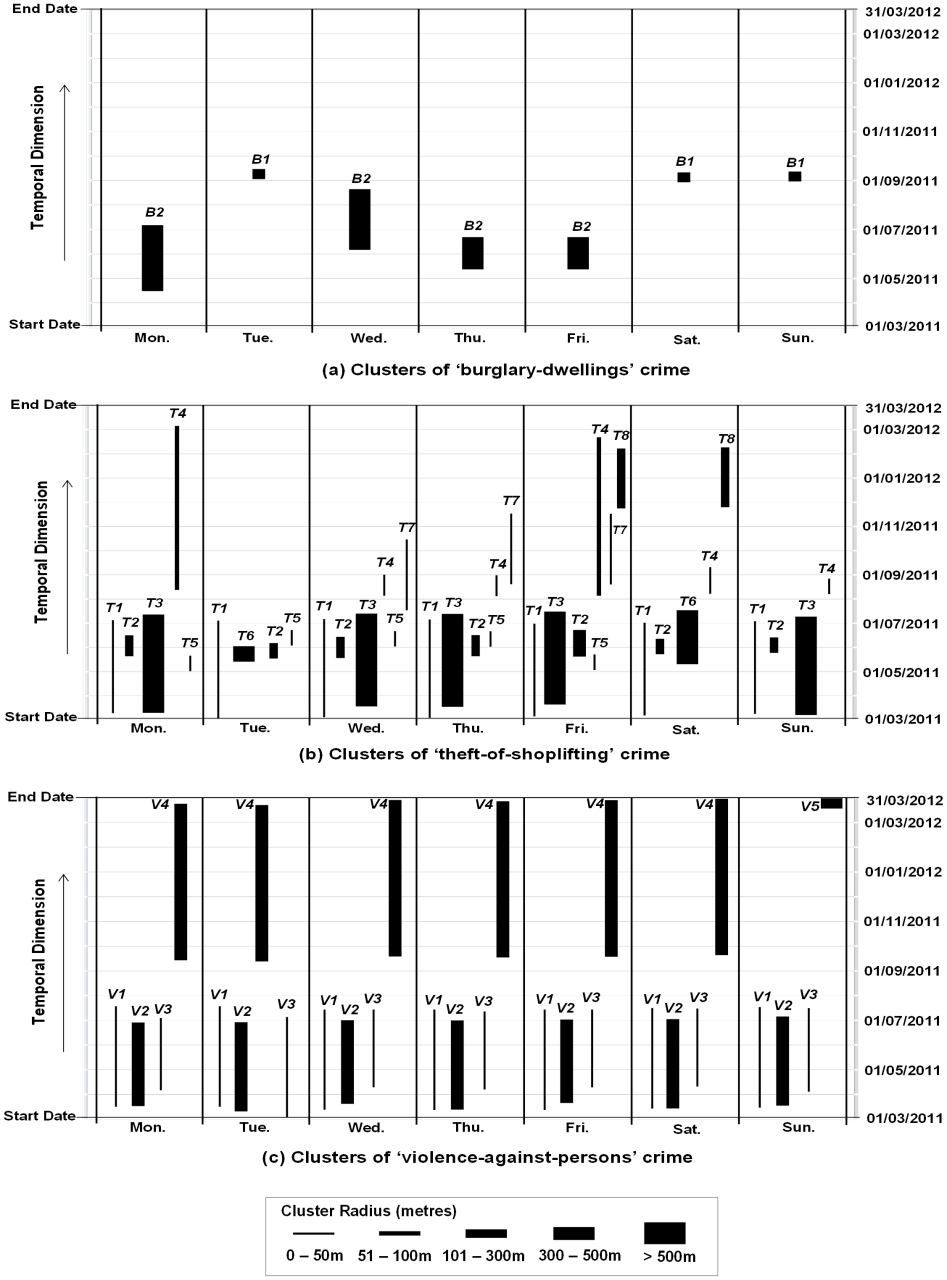
Temporal Segmentation Effects (a) “Burglary-dwellings” crime clusters (b) “Theft-of-shoplifting” crime clusters (c) “Violence-against-persons” crime clusters.

Figures 5a, 5b and 5c show the clusters detected at seven temporal segmentations for the “burglary-dwellings”, “theft-of-shoplifting” and “violence-against-persons” crime types respectively.

In the “burglary-dwellings” crime dataset (Figure 5a), Cluster *B1* is detected at two segmentations i.e. Tuesday and Saturday segmentations, with similar values for ‘start date’, spatial extent and temporal duration. Cluster *B2* is detected at four segmentations, i.e. Monday, Wednesday, Thursday and Friday, with different ‘start dates’ and temporal durations, except that the results on Thursday and Friday are similar. No cluster is detected with Sunday segmentation. The cluster with the next smallest p-value for the Sunday segmentation is Cluster *B1*, with a p-value as 0.056 (see Table S1-b in Appendix S1). This implies that *B1* is a week cluster, and this type of crime displays weekly patterns. “Burglary-dwellings” crime is more likely to occur on working days (Monday to Friday). This also may imply that on other days, Area *B2* might have higher chance of being burglarised. Further details of these clusters can be found at Table S1-b in Appendix S1.

The “theft-of-shoplifting” dataset featured more clusters and equally showed more variations in the clusters detected at all segmentations (Figure 5b). Only Clusters *T1* and *T2* appear at all segmentations while others are not consistent at all segmentations. Therefore, the number of clusters detected changes with the segmentation. For example, four clusters are detected with Tuesday and Sunday segmentations, five with Monday and Saturday segmentations, six with Wednesday and Thursday and seven with Friday segmentation. The varying number of clusters seems to reflect daily shopping patterns, although further clarification is needed on this aspect. Further details of these clusters can be found at Table S2-b in Appendix S1.

The “violence-against-persons” dataset showed the most consistent results (Figure 5c). The clusters barely changed except in the Sunday segmentation. In this case, Cluster *V4* is not detected but an additional cluster, *V5*, is detected which overlaps temporally with Cluster *V4*. The consistent results reflect the fact that ‘violence-again-persons’ includes domestic violence, which does not have specific daily patterns. Further details of these clusters can be found at Table S3-b in Appendix S1.

In summary, the segmentation of the dataset alters the clustering results depending on the crime type, which suggests different crime types have their own cycles during a week. “Burglary-dwellings” is relatively highly high on other days except on Sunday. “Theft-of-shoplifting” is relatively quiet on Sunday (this might be due to the short opening hours), and relatively busier on Wednesday to Friday. There are no daily patterns in the “violence-against-persons” crime type. Also, the consistency of some clusters suggests that certain clusters can be considered as being stronger than the others, and therefore provides clues to which clusters can be considered as ‘true’ clusters, e.g. Clusters *T1* and *T2* for the “theft-of-shoplifting”, and Clusters *V1*, *V2*, and *V3* for the “violence-against-persons”.

### 4.3 Effect of Change in Temporal Boundary

For each crime type, same adjustments are made to the temporal length of the daily dataset as follows;

- March1, 2011–March 31, 2012 (Boundary *A*, original boundary)
- April 1, 2011–March 31, 2012 (Boundary *B*)
- March 1, 2011–Feb 29, 2012 (Boundary *C*)
- April 1, 2011–Feb 29, 2012 (Boundary *D*)

**Figure 6:**
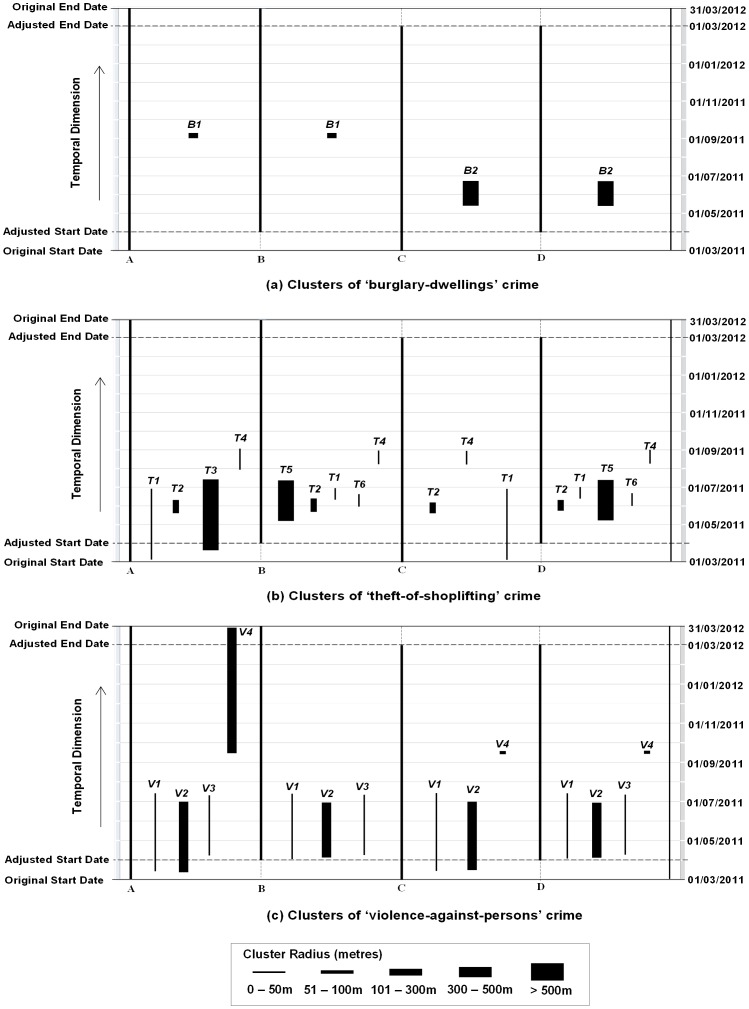
Temporal Boundary Effects (a) “Burglary-dwellings” crime clusters (b) “Theft-of-shoplifting” crime clusters (c) “Violence-against-persons” crime clusters.

All clusters detected in each boundary are placed on the right hand-side of their respective boundary and are arranged in order of statistical significance from left to right as shown in Figures 6a, 6b and 6c for the three crimes respectively.

In the “burglary-dwellings” dataset, the adjustments to the temporal length had a significant influence on the clusters detected (Figure 6a). Only 2 clusters, *B1* and *B2*, were identified as significant at the original length *A* and the adjusted length D respectively. The most significant clusters in the other two boundaries *B* and *C*, are clusters *B1* and *B2*, respectively, with p-values of 0.071 and 0.055 respectively. This suggests that these clusters may not be ‘true’ clusters given their relatively low p-values (compared with the threshold value of 0.05), and their inconsistency across different boundaries. Therefore, it is difficult to be certain about which cluster is a ‘true’ hotspot of “burglary-dwellings”. Further details of these clusters can be found at Table S1-c in Appendix S1.

In the “theft-of-shoplifting” crime type (Figure 6b), Clusters *T2* and *T4* are very consistent in their desriptions, Cluster *T1* is also detected at all 4 boundary ranges, but has two durations, one for Cases A and C, and one for Cases B and D. Clusters *T3*, T5 and *T6* appear at different boundary ranges, but are not consistent. Therefore, Clusters *T1*, *T2* and *T4* can be considered “true” clusters. Further details of these clusters can be found at Table S2-c in Appendix S1.

Again, boundary effects are relatively less obvious on the clusters detected for the “violence-against-persons” crime type, e.g. clusters *V1* and *V2* (Figure 6c). Both Clusters *V3* and *V4* appear in 3 but not 4 cases. So we may consider Clusters *V1* and *V2* in Figure 6c as “true” clusters based on their consistency. Further details of these clusters can be found at Table S3-c in Appendix S1.

### 4.4 Further Discussion

For comparison purposes, we put the results of one crime type in one table (see Appendix S1 Tables S1–S3), with each effect in a sub-table (a, b, c) for aggregation, segmentation and boundary. We can see that there is no cluster appearing consistently in all the scales for “burglary-dwellings”. For “theft-of-shopping”, Clusters *T1* and *T2* are significant at all scales, as are the Clusters *V1* and *V2* for ‘violence-against-persons’. These are the ‘true’ hotspots.

If we examine the p-values more carefully, we will find that most clusters that are consistent across all scales have p-values smaller than 0.005. This applies to the 4 clusters that we have identified above. This means that a p-value of 0.005 could be used to identify ‘true’ clusters that are consistent across scales, and to mitigate the MTUP effect.

More clusters are found at coarse intervals, which imply that we can use aggregation to detect significant clusters more quickly, given much less data than are required to process at a coarse scale. Although the exact temporal duration of clusters identified at two scales might be different, the clusters detected at the coarse scale could be used as a guide for further analysis. This will significantly improve the processing speed.

Furthermore, we can use segmentation to probe the cyclic patterns of crime varying within the week. The results demonstrate that “burglary-dwellings” is generally quiet on weekends and Tuesday (no hotspot on Sunday, and very short duration on Saturday and Tuesday); “Theft-of-shopping” is relatively quiet on Tuesday and Sunday compared with other days, and Friday is very active with highest number and longest duration of the hotspots; and “violence-against-persons” has no time varying patterns at all.

## Conclusions and Future work

This study investigated the impact of temporal effects that are usually ignored in space-time analysis. We formally defined the MTUP (modifiable temporal unit problem) as a consisting of temporal aggregation, segmentation and boundary effects. In our experiment, we examined these effects on space-time cluster detection of crime hotspots using space-time scan statistics (STSS). In general, there is tendency to detect different clusters as the aggregation, segmentation and boundary of a space-time dataset are altered. This means that we should be cautious when we use a particular temporal scale, segmentation and boundary for analysis. But the most significant clusters (‘true’ clusters) with a p-value smaller than 0.005 can be consistently detected no matter the temporal configuration of the dataset.

We have discussed how to use these three temporal effects to improve the efficiency of STSS and to gain insight into crime patterns. Aggregation could be used to find the significant clusters much more quickly than at lower scales; segmentation could be used to understand the cyclic patterns of crime types. Further experiments on other crime types and on other data sets (such as health data) will be needed to examine if the results can be generalised to other applications.

In the next phase of this study, we will consider the “Modifiable Spatio-Temporal Unit Problem” (MSTUP) as the intersection of MAUP and MTUP. The question of how to select the most suitable spatial and/or temporal scales for space-time cluster detection is an area that also needs further research. Also, an extension of this study to other techniques such as space-time clustering using technique like space-time kernel density estimation will be carried out in the future.

## Supporting Information

Appendix S1 Contains the following files: Table 1: Cluster detection for “Burglary-dwellings”. Table 2: Cluster detection for “theft-of-shoplifting”. Table 3: Cluster detection for “violence-against-persons”.
(DOCX)
