# Supplementary material for: Modifiable Temporal Unit Problem (MTUP) and Its Effect on Space-Time Cluster Detection
Source: PLoS One. 2014 Jun 27;9(6):e100465. doi: 10.1371/journal.pone.0100465 (PMC4074055; doi:10.1371/journal.pone.0100465)
Supplement: Appendix S1 — Contains the following files: Table 1: Cluster detection for “Burglary-dwellings”. Table 2: Cluster detection for “theft-of-shoplifting”. Table 3: Cluster detection for “violence-against-persons”. (DOCX) [file pone.0100465.s001.docx]

**Appendix S1 -** **Cluster detection for 'Burglary-dwelling'**

| Table S1-a: aggregation effects | | |  | | |  |  | |  | |  | | |  |
| --- | --- | --- | --- | --- | --- | --- | --- | --- | --- | --- | --- | --- | --- | --- |
| Temporal Aggregation | Cluster ID | X | | Y | Radius(m) | | | Start_Date | | End_Date | | P_Value |  |  |
| Daily | B1 | 527375 | | 185375 | 303 | | | 30/08/2011 | | 08/09/2011 | | 0.036 |  |  |
| Weekly | B2 | 529624 | | 182625 | 1000 | | | 11/04/2011 | | 03/07/2011 | | 0.031 |  |  |
| Monthly | B3 | 530875 | | 182125 | 559 | | | 01/05/2011 | | 31/07/2011 | | 0.009 |  |  |

| Table S1-b: segmentation effects | | | |  | |  | |  | |  | |  | |
| --- | --- | --- | --- | --- | --- | --- | --- | --- | --- | --- | --- | --- | --- |
| Starting Day of Weekly Segmentation | Cluster ID | X | Y | | Radius(m) | | Start_Date | | End_Date | | P_Value | |  |
| Monday | B2 | 529624 | 182625 | | 1000 | | 11/04/2011 | | 03/07/2011 | | 0.031 | |  |
| Tuesday | B1 | 527375 | 185375 | | 303 | | 30/08/2011 | | 12/09/2011 | | 0.037 | |  |
| Wednesday | B2 | 529875 | 182625 | | 902 | | 06/04/2011 | | 21/06/2011 | | 0.041 | |  |
| Thursday | B2 | 529875 | 182625 | | 901 | | 12/05/2011 | | 22/06/2011 | | 0.029 | |  |
| Friday | B2 | 529875 | 182375 | | 791 | | 13/05/2011 | | 23/06/2011 | | 0.016 | |  |
| Saturday | B1 | 527375 | 185375 | | 303 | | 27/08/2011 | | 09/09/2011 | | 0.041 | |  |
| Sunday | B1 | 527375 | 185375 | | 303 | | 28/08/2011 | | 10/09/2011 | | 0.056 | |  |

Table S1-c: boundary effects

| Temporal Length | Cluster ID | X | Y | Radius(m) | Start_Date | End_Date | P_Value |
| --- | --- | --- | --- | --- | --- | --- | --- |
| A | B1 | 527375 | 185375 | 303 | 30/08/2011 | 08/09/2011 | 0.036 |
| B | B1 | 527375 | 185375 | 303 | 30/08/2011 | 08/09/2011 | 0.071 |
| C | B2 | 529875 | 182375 | 791 | 13/05/2011 | 23/06/2011 | 0.055 |
| D | B2 | 529875 | 182375 | 791 | 13/05/2011 | 23/06/2011 | 0.042 |

**Appendix S2 - Cluster detection for ‘Theft-shoplifting’**

Table S2-a: aggregation effects

| Temporal aggregation | Cluster ID | X | Y | Radius(m) | Start_Date | End_Date | P_Value |
| --- | --- | --- | --- | --- | --- | --- | --- |
| Daily | T1 | 530125 | 181125 | 0 | 05/03/2011 | 30/06/2011 | 0.001 |
|  | T2 | 526625 | 185625 | 250 | 23/05/2011 | 14/06/2011 | 0.002 |
|  | T3 | 528235 | 184817 | 668 | 18/03/2011 | 13/07/2011 | 0.006 |
|  | T4 | 530188 | 181116 | 41 | 07/08/2011 | 30/08/2011 | 0.008 |
| Weekly | T1 | 530125 | 181125 | 0 | 07/03/2011 | 03/07/2011 | 0.002 |
|  | T2 | 526625 | 185625 | 250 | 23/05/2011 | 19/06/2011 | 0.002 |
|  | T3 | 528235 | 184817 | 668 | 07/03/2011 | 10/07/2011 | 0.004 |
|  | T4 | 530184 | 181225 | 114 | 08/08/2011 | 04/03/2012 | 0.039 |
|  | T5 | 528875 | 183875 | 0 | 30/05/2011 | 19/06/2011 | 0.043 |
| Monthly | T1 | 530125 | 181125 | 0 | 01/03/2011 | 30/06/2011 | 0.001 |
|  | T3 | 528125 | 184875 | 791 | 01/03/2011 | 31/07/2011 | 0.001 |
|  | T4 | 530188 | 181116 | 41 | 01/08/2011 | 31/08/2011 | 0.001 |
|  | T6 | 529142 | 183744 | 262 | 01/12/2011 | 31/01/2012 | 0.001 |
|  | T7 | 528625 | 184125 | 250 | 01/10/2011 | 31/03/2012 | 0.032 |
|  | T5 | 528875 | 183875 | 0 | 01/03/2011 | 30/06/2011 | 0.032 |
|  | T2 | 526625 | 185625 | 0 | 01/05/2011 | 30/06/2011 | 0.042 |

Table S2-b: segmentation effects

| Starting Day of Weekly Segmentation | Cluster ID | X | Y | Radius(m) | Start_Date | End_Date | P_Value |
| --- | --- | --- | --- | --- | --- | --- | --- |
|  |  |  |  |  |  |  |  |
| MONDAY | T1 | 530125 | 181125 | 0 | 07/03/2011 | 03/07/2011 | 0.002 |
|  | T2 | 526625 | 185625 | 250 | 23/05/2011 | 19/06/2011 | 0.002 |
|  | T3 | 528235 | 184817 | 668 | 07/03/2011 | 10/07/2011 | 0.004 |
|  | T4 | 530184 | 181225 | 114 | 08/08/2011 | 04/03/2012 | 0.039 |
|  | T5 | 528875 | 183875 | 0 | 30/05/2011 | 19/06/2011 | 0.043 |
| TUESDAY | T1 | 530125 | 181125 | 0 | 01/03/2011 | 04/07/2011 | 0.001 |
|  | T6 | 527625 | 184625 | 559 | 10/05/2011 | 30/05/2011 | 0.001 |
|  | T2 | 526625 | 185625 | 250 | 17/05/2011 | 06/06/2011 | 0.009 |
|  | T5 | 528875 | 183875 | 0 | 31/05/2011 | 20/06/2011 | 0.03 |
| WEDNESDAY | T1 | 530125 | 181125 | 0 | 02/03/2011 | 05/07/2011 | 0.001 |
|  | T2 | 526625 | 185625 | 250 | 18/05/2011 | 14/06/2011 | 0.001 |
|  | T3 | 528235 | 184817 | 668 | 16/03/2011 | 12/07/2011 | 0.001 |
|  | T4 | 530188 | 181116 | 41 | 03/08/2011 | 30/08/2011 | 0.012 |
|  | T5 | 528875 | 183875 | 0 | 01/06/2011 | 21/06/2011 | 0.034 |
|  | T7 | 526385 | 185753 | 0 | 17/08/2011 | 15/11/2011 | 0.045 |
| THURSDAY | T1 | 530125 | 181125 | 0 | 03/03/2011 | 06/07/2011 | 0.002 |
|  | T3 | 528235 | 184817 | 668 | 17/03/2011 | 13/07/2011 | 0.002 |
|  | T2 | 526625 | 185625 | 250 | 19/05/2011 | 15/06/2011 | 0.003 |
|  | T5 | 528875 | 183875 | 0 | 02/06/2011 | 22/06/2011 | 0.02 |
|  | T4 | 530188 | 181116 | 41 | 04/08/2011 | 31/08/2011 | 0.026 |
|  | T7 | 526385 | 185753 | 0 | 18/08/2011 | 16/11/2011 | 0.037 |
| FRIDAY | T1 | 530125 | 181125 | 0 | 04/03/2011 | 30/06/2011 | 0.001 |
|  | T3 | 528235 | 184817 | 668 | 18/03/2011 | 14/07/2011 | 0.001 |
|  | T2 | 526685 | 185626 | 315 | 20/05/2011 | 23/06/2011 | 0.001 |
|  | T5 | 528875 | 183875 | 0 | 03/06/2011 | 23/06/2011 | 0.014 |
|  | T4 | 530184 | 181225 | 114 | 05/08/2011 | 23/02/2012 | 0.015 |
|  | T7 | 526385 | 185753 | 0 | 19/08/2011 | 17/11/2011 | 0.023 |
|  | T8 | 529142 | 183744 | 262 | 25/11/2011 | 09/02/2012 | 0.042 |
| SATURDAY | T1 | 530125 | 181125 | 0 | 05/03/2011 | 01/07/2011 | 0.002 |
|  | T2 | 526625 | 185625 | 250 | 21/05/2011 | 10/06/2011 | 0.004 |
|  | T6 | 527625 | 184625 | 559 | 07/05/2011 | 15/07/2011 | 0.004 |
|  | T4 | 530262 | 181177 | 96 | 06/08/2011 | 09/09/2011 | 0.017 |
|  | T8 | 529142 | 183744 | 262 | 26/11/2011 | 10/02/2012 | 0.046 |
| SUNDAY | T1 | 530125 | 181125 | 0 | 06/03/2011 | 02/07/2011 | 0.002 |
|  | T2 | 526625 | 185625 | 250 | 22/05/2011 | 11/06/2011 | 0.002 |
|  | T3 | 528235 | 184817 | 668 | 06/03/2011 | 09/07/2011 | 0.002 |
|  | T4 | 530188 | 181116 | 41 | 07/08/2011 | 27/08/2011 | 0.003 |

Table S2-c: boundary effects

| Temporal Length | Cluster ID | X | Y | Radius(m) | Start_Date | End_Date | P_Value |
| --- | --- | --- | --- | --- | --- | --- | --- |
| A | T1 | 530125 | 181125 | 0 | 05/03/2011 | 30/06/2011 | 0.001 |
|  | T2 | 526625 | 185625 | 250 | 23/05/2011 | 14/06/2011 | 0.002 |
|  | T3 | 528235 | 184817 | 668 | 18/03/2011 | 13/07/2011 | 0.006 |
|  | T4 | 530188 | 181116 | 41 | 07/08/2011 | 30/08/2011 | 0.008 |
| B | T5 | 527625 | 184625 | 559 | 07/05/2011 | 13/07/2011 | 0.003 |
|  | T2 | 526625 | 185625 | 250 | 23/05/2011 | 14/06/2011 | 0.009 |
|  | T1 | 530125 | 181125 | 0 | 11/06/2011 | 30/06/2011 | 0.009 |
|  | T6 | 528875 | 183875 | 0 | 31/05/2011 | 21/06/2011 | 0.01 |
|  | T4 | 530188 | 181116 | 41 | 07/08/2011 | 30/08/2011 | 0.04 |
| C | T2 | 526625 | 185625 | 250 | 23/05/2011 | 10/06/2011 | 0.006 |
|  | T4 | 530188 | 181116 | 41 | 07/08/2011 | 30/08/2011 | 0.008 |
|  | T1 | 530125 | 181125 | 0 | 05/03/2011 | 30/06/2011 | 0.009 |
| D | T2 | 526625 | 185625 | 250 | 23/05/2011 | 10/06/2011 | 0.006 |
|  | T1 | 530125 | 181125 | 0 | 11/06/2011 | 30/06/2011 | 0.008 |
|  | T5 | 527625 | 184625 | 559 | 07/05/2011 | 13/07/2011 | 0.011 |
|  | T6 | 528875 | 183875 | 0 | 31/05/2011 | 21/06/2011 | 0.013 |
|  | T4 | 530188 | 181116 | 41 | 07/08/2011 | 30/08/2011 | 0.021 |

**Appendix S3 - Cluster detection for ‘Violence-against-person’**

Table S3-a: aggregation effects

| Temporal Aggregation | Cluster ID | X | Y | Radius(m) | Start_Date | End_Date | P_Value |
| --- | --- | --- | --- | --- | --- | --- | --- |
|  |  |  |  |  |  |  |  |
| Daily | V1 | 529375 | 182125 | 0 | 13/03/2011 | 14/07/2011 | 0.001 |
|  | V2 | 527875 | 184375 | 354 | 10/03/2011 | 29/06/2011 | 0.002 |
|  | V3 | 528875 | 183875 | 0 | 09/04/2011 | 13/07/2011 | 0.033 |
|  | V4 | 528518 | 184071 | 316 | 14/09/2011 | 30/03/2012 | 0.04 |
| Weekly | V1 | 529375 | 182125 | 0 | 14/03/2011 | 17/07/2011 | 0.001 |
|  | V2 | 527875 | 184375 | 354 | 14/03/2011 | 26/06/2011 | 0.001 |
|  | V3 | 528875 | 183875 | 0 | 04/04/2011 | 03/07/2011 | 0.004 |
|  | V4 | 528518 | 184071 | 316 | 12/09/2011 | 25/03/2012 | 0.022 |
| Monthly | V1 | 529625 | 182125 | 250 | 01/03/2011 | 31/05/2011 | 0.001 |
|  | V2 | 527875 | 184375 | 354 | 01/03/2011 | 30/06/2011 | 0.001 |
|  | V3 | 528873 | 183552 | 323 | 01/03/2011 | 30/06/2011 | 0.002 |
|  | V4 | 528495 | 184298 | 122 | 01/03/2012 | 31/03/2012 | 0.008 |
|  | V5 | 528875 | 184125 | 249 | 01/09/2011 | 30/11/2011 | 0.033 |

Table S3-b: segmentation effects

| Starting Day of Weekly Segmentation | Cluster ID | X | Y | Radius(m) | Start_Date | End_Date | P_Value |
| --- | --- | --- | --- | --- | --- | --- | --- |
| Monday | V1 | 529375 | 182125 | 0 | 14/03/2011 | 17/07/2011 | 0.001 |
|  | V2 | 527875 | 184375 | 354 | 14/03/2011 | 26/06/2011 | 0.001 |
|  | V3 | 528875 | 183875 | 0 | 04/04/2011 | 03/07/2011 | 0.004 |
|  | V4 | 528518 | 184071 | 316 | 12/09/2011 | 25/03/2012 | 0.022 |
| Tuesday | V1 | 529375 | 182125 | 0 | 15/03/2011 | 18/07/2011 | 0.001 |
|  | V2 | 527875 | 184375 | 354 | 08/03/2011 | 27/06/2011 | 0.001 |
|  | V4 | 528518 | 184071 | 316 | 13/09/2011 | 26/03/2012 | 0.008 |
|  | V3 | 528875 | 183875 | 0 | 01/03/2011 | 04/07/2011 | 0.015 |
| Wednesday | V1 | 529375 | 182125 | 0 | 11/03/2011 | 14/07/2011 | 0.001 |
|  | V2 | 527875 | 184375 | 354 | 18/03/2011 | 30/06/2011 | 0.001 |
|  | V3 | 528875 | 183875 | 0 | 08/04/2011 | 14/07/2011 | 0.003 |
|  | V4 | 528518 | 184071 | 316 | 16/09/2011 | 29/03/2012 | 0.032 |
| Thursday | V1 | 529375 | 182125 | 0 | 10/03/2011 | 13/07/2011 | 0.001 |
|  | V2 | 527875 | 184375 | 354 | 10/03/2011 | 29/06/2011 | 0.001 |
|  | V3 | 528875 | 183875 | 0 | 07/04/2011 | 13/07/2011 | 0.002 |
|  | V4 | 528518 | 184071 | 316 | 15/09/2011 | 28/03/2012 | 0.008 |
| Friday | V1 | 529375 | 182125 | 0 | 11/03/2011 | 14/07/2011 | 0.001 |
|  | V2 | 527875 | 184375 | 354 | 18/03/2011 | 30/06/2011 | 0.001 |
|  | V3 | 528875 | 183875 | 0 | 08/04/2011 | 14/07/2011 | 0.003 |
|  | V4 | 528518 | 184071 | 316 | 16/09/2011 | 29/03/2012 | 0.032 |
| Saturday | V1 | 529375 | 182125 | 0 | 12/03/2011 | 15/07/2011 | 0.001 |
|  | V2 | 527875 | 184375 | 354 | 12/03/2011 | 01/07/2011 | 0.002 |
|  | V3 | 528875 | 183875 | 0 | 09/04/2011 | 15/07/2011 | 0.004 |
|  | V4 | 528518 | 184071 | 316 | 17/09/2011 | 30/03/2012 | 0.021 |
| Sunday | V1 | 529375 | 182125 | 0 | 13/03/2011 | 16/07/2011 | 0.001 |
|  | V2 | 527875 | 184375 | 354 | 13/03/2011 | 02/07/2011 | 0.001 |
|  | V3 | 528875 | 183875 | 0 | 03/04/2011 | 16/07/2011 | 0.005 |
|  | V5 | 528711 | 184563 | 546 | 18/03/2012 | 31/03/2012 | 0.044 |

Table S3-c: boundary effects

| Temporal Length | Cluster ID | X | Y | Radius(m) | Start_Date | End_Date | P_Value |
| --- | --- | --- | --- | --- | --- | --- | --- |
| A | V1 | 529375 | 182125 | 0 | 13/03/2011 | 14/07/2011 | 0.001 |
|  | V2 | 527875 | 184375 | 354 | 10/03/2011 | 29/06/2011 | 0.002 |
|  | V3 | 528875 | 183875 | 0 | 09/04/2011 | 13/07/2011 | 0.033 |
|  | V4 | 528518 | 184071 | 316 | 14/09/2011 | 30/03/2012 | 0.04 |
| B | V1 | 529375 | 182125 | 0 | 02/04/2011 | 14/07/2011 | 0.002 |
|  | V2 | 527875 | 184375 | 354 | 03/04/2011 | 29/06/2011 | 0.002 |
|  | V3 | 528875 | 183875 | 0 | 09/04/2011 | 13/07/2011 | 0.008 |
| C | V1 | 529375 | 182125 | 0 | 13/03/2011 | 14/07/2011 | 0.002 |
|  | V2 | 527875 | 184375 | 354 | 13/03/2011 | 29/06/2011 | 0.003 |
|  | V3 | 528645 | 184143 | 181 | 14/09/2011 | 19/09/2011 | 0.034 |
| D | V1 | 529375 | 182125 | 0 | 02/04/2011 | 14/07/2011 | 0.003 |
|  | V2 | 527875 | 184375 | 354 | 03/04/2011 | 29/06/2011 | 0.004 |
|  | V3 | 528875 | 183875 | 0 | 09/04/2011 | 13/07/2011 | 0.021 |
|  | V4 | 528645 | 184143 | 181 | 14/09/2011 | 19/09/2011 | 0.048 |
